# Self-Inflicted Abdominal Air Insufflation Leading to Diffuse Subcutaneous Emphysema, Pneumoperitoneum, Pneumomediastinum, Pneumopericardium, and Pneumothorax: A Case Report

**DOI:** 10.7759/cureus.30278

**Published:** 2022-10-13

**Authors:** Anthony J Corsi, Ahmed Soukat Ali, Dinesh Kumar

**Affiliations:** 1 Medicine, Upstate University Hospital, Syracuse, USA

**Keywords:** pneumothorax, pneumopericardium, pneumomediastinum, pneumoperitoneum, subcutaneous emphysema

## Abstract

Subcutaneous emphysema is defined by air becoming confined in the soft tissues beneath the skin and it may occur following various surgical procedures and specific penetrating trauma. While treatment is typically conservative and not required in most cases, massive subcutaneous emphysema may cause significant morbidity and sometimes life-threatening complications such as tension pneumomediastinum and respiratory compromise. Notably, no instances of self-inflicted air insufflation into the abdominal cavity have been reported in the literature. This report depicts a case of a self-inflicted air insufflation in a 40-year-old man via penetration of his umbilicus with a manual air compressor leading to widespread subcutaneous emphysema, pneumoperitoneum, pneumomediastinum, pneumopericardium, and pneumothorax. The pathway of possible air movement between body cavities has been theorized, but excluding case reports and anecdotal evidence, treatment of severe subcutaneous emphysema is less clear. This case report intends to record this unique instance of extensive subcutaneous emphysema and to emphasize the necessity for more definitive guidelines in managing these patients.

## Introduction

Subcutaneous emphysema refers to air becoming trapped under the skin and infiltrating the soft tissues, typically of the neck and chest. It can manifest as a complication from specific surgical procedures (e.g., coronary artery bypass surgery, lobectomy, tracheostomy, etc.), or arise secondary to penetrating trauma, infection, or malignancy [1]. In most cases, subcutaneous emphysema is harmless and does not require treatment. However, the reported sensation of skin tension may arise if severe, and symptoms such as dysphonia and dysphagia can develop alongside concurrent mediastinal emphysema. If the deeper tissues of the chest or abdomen are affected, restricted lung expansion can even cause life-threatening respiratory compromise [1-3]. The air within the abdominal cavity may also lead to the development of air emboli via the introduction of air into the abdominal blood vessels or organs, possibly causing neurological damage or cardiovascular collapse [4]. Pneumomediastinum, pneumopericardium, and pneumoperitoneum may also occur in some scenarios as the fascia allows the passage of air between cavities [5-7]. No cases of intentional body insufflation have been documented in the literature to date. In this case report, we describe a 40-year-old man who injected air through his umbilicus leading to extensive subcutaneous emphysema, pneumoperitoneum, pneumomediastinum, pneumopericardium, and pneumothorax.

## Case presentation

**Figure 1:**
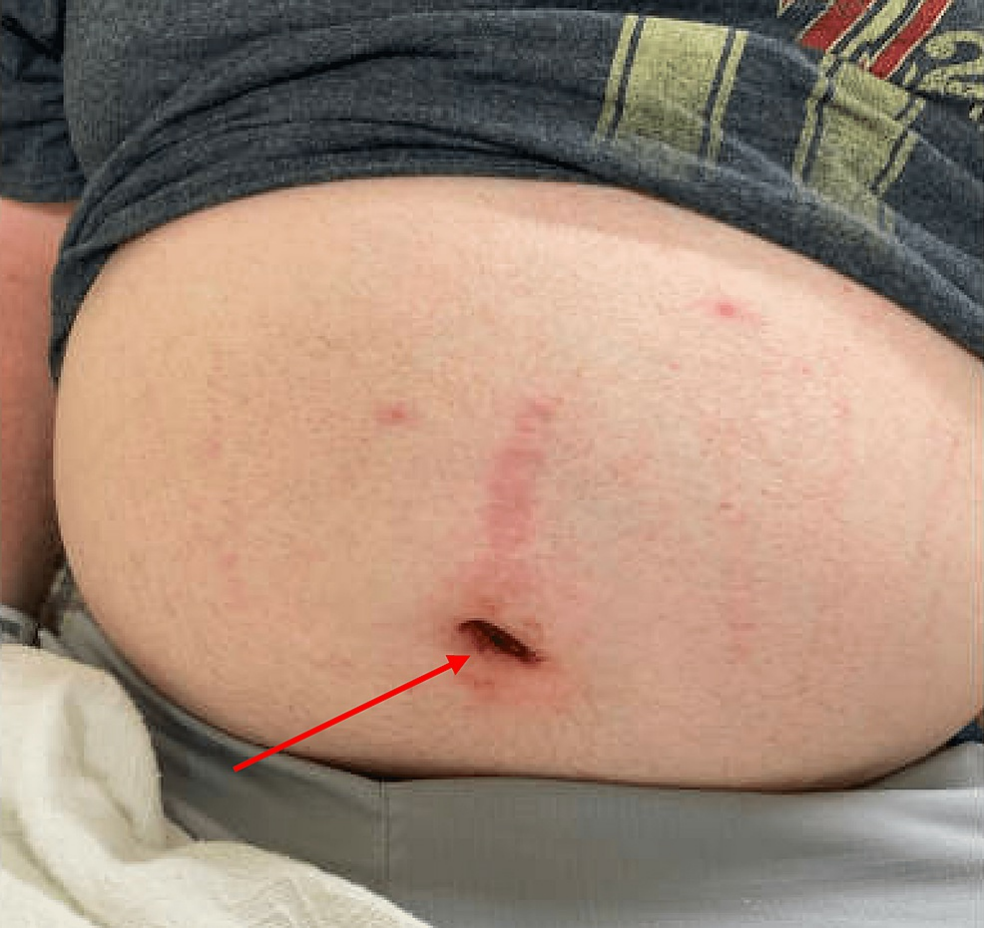
Markedly distended abdomen upon presentation. The red arrow indicates the site of abdominal penetration.

A 40-year-old male presented to the emergency department with shortness of breath and tightness around his chest and neck after using a manual air compressor to inflate air through his umbilicus. This patient notably endorsed a past medical history of schizophrenia, bipolar disorder, and autism spectrum disorder. He had also reported a history of severe compulsions that resulted in him doing this in the past to a lesser degree. The patient later confirmed during his hospital admission that he completed this act as a form of sexual gratification. Upon arrival, the patient was hemodynamically stable and saturating well on room air. Widespread subcutaneous emphysema with palpable crepitus extending from the abdomen to the periorbital region was apparent on physical exam. His face was swollen, and his abdomen distended. A mark with dried blood appeared over the umbilicus (Figure 1).

**Figure 2:**
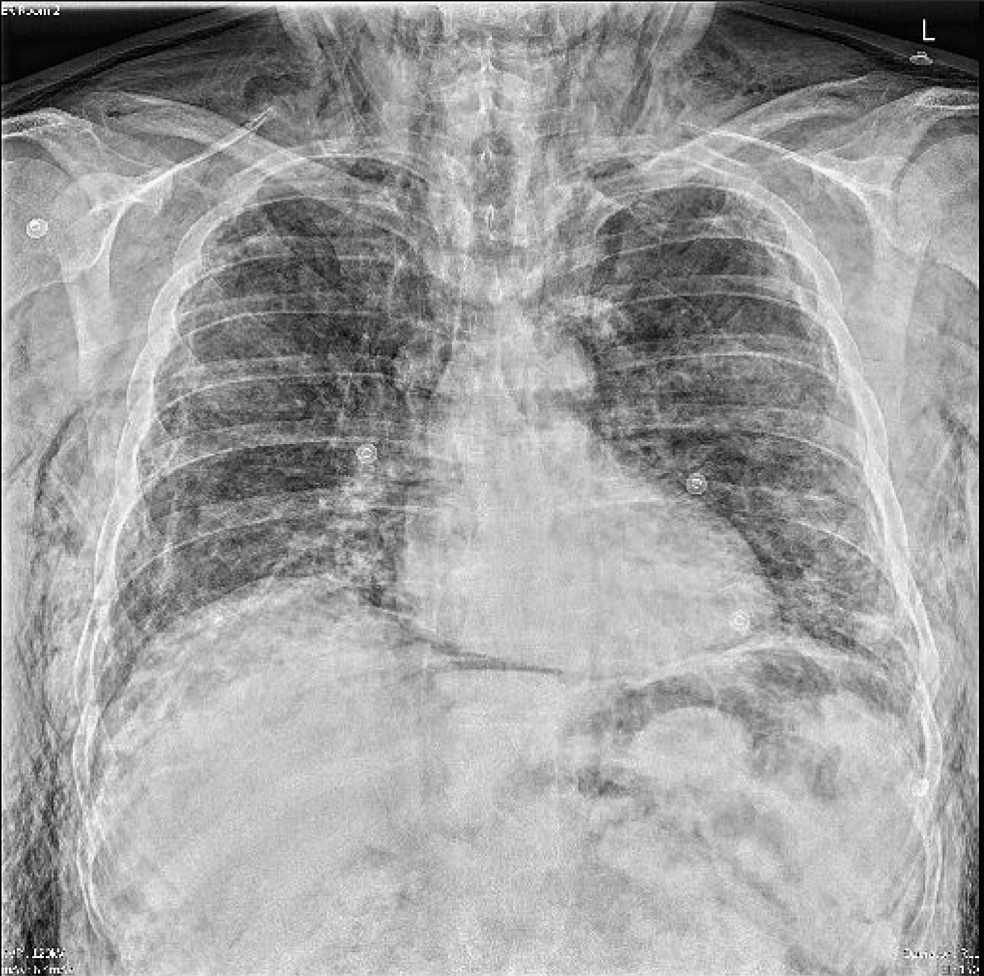
Chest X-ray demonstrating diffuse subcutaneous emphysema and pneumoperitoneum.

**Figure 3:**
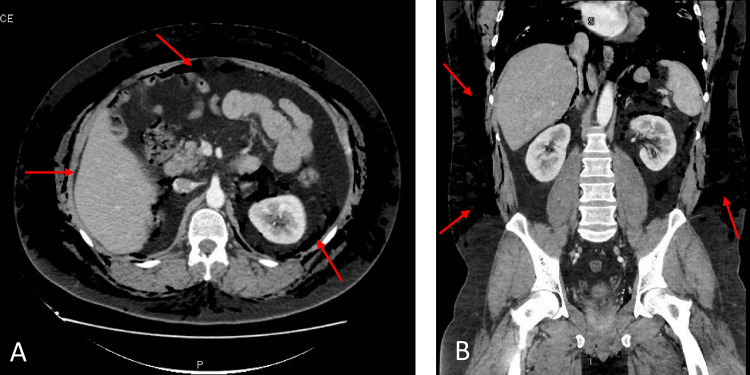
Axial (A) and coronal (B) views of CT abdomen demonstrating pneumoperitoneum and diffuse subcutaneous emphysema. The red arrows indicate the presence of free air.

**Figure 4:**
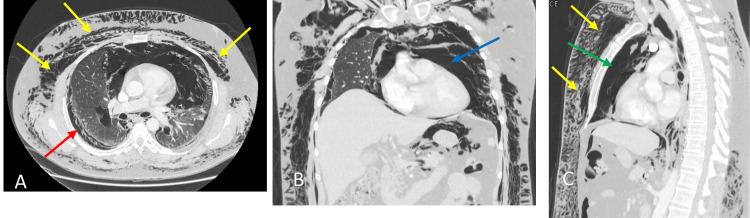
Axial (A), coronal (B), and sagittal (C) views of CT thorax demonstrating subcutaneous emphysema (yellow arrows), pneumothorax (red arrow), pneumopericardium (blue arrow), and pneumomediastinum (green arrow).

**Figure 5:**
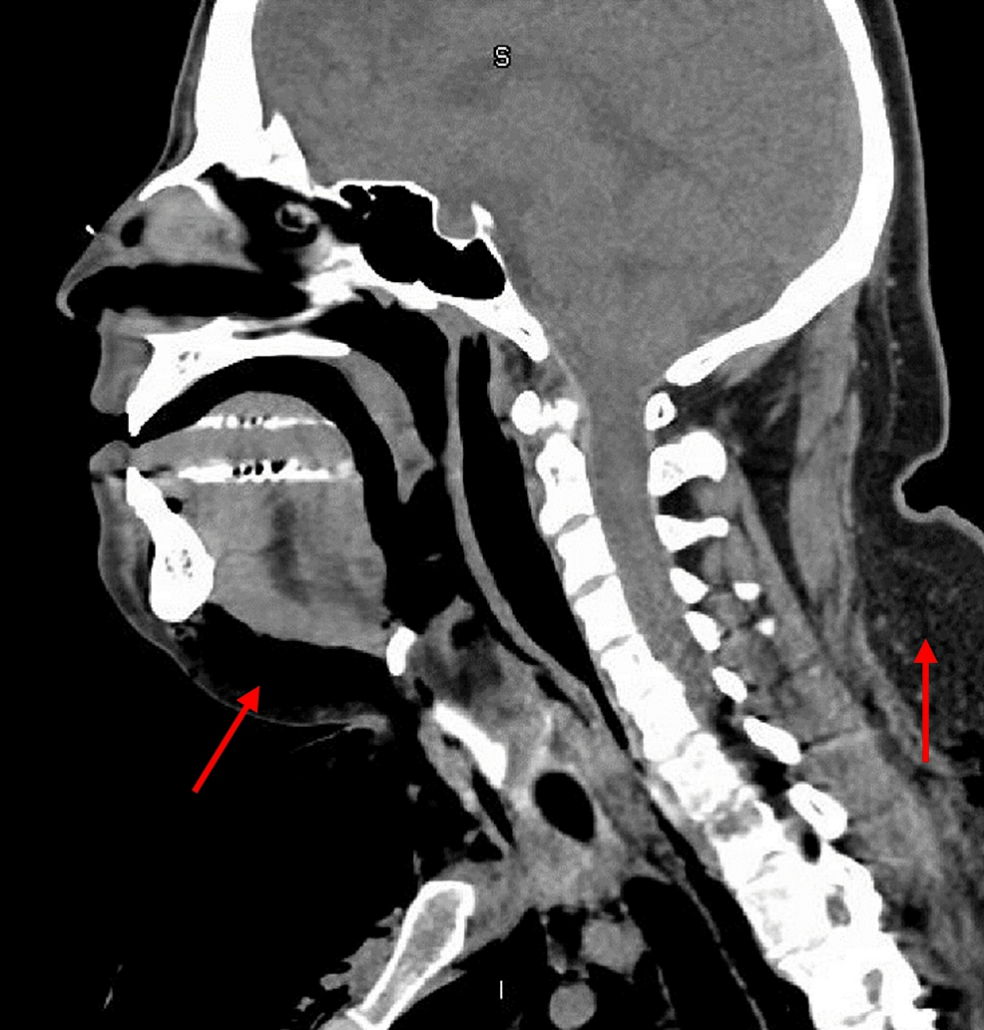
Sagittal view of CT neck indicating the extension of subcutaneous emphysema (red arrows).

Basic metabolic profile and complete blood count were unremarkable excluding a mild white blood cell count elevation of 14.3/cumm. Electrocardiogram and troponin levels were both unremarkable as well. Chest X-ray revealed prominent subcutaneous emphysema throughout the chest and neck with evidence of pneumomediastinum. The CT of the neck, thorax, and abdomen further demonstrated subcutaneous emphysema from the superior thorax through the temporal and periorbital areas, as well as a large pneumomediastinum, pneumopericardium, bilateral small pneumothoraces, and moderate retroperitoneal emphysema (Figures 2-5). General surgery, cardiac surgery, and thoracic surgery services were consulted in the emergency department, but all recommended that no acute surgical interventions were indicated. The patient was subsequently admitted to the general medicine service for further observation.

The patient’s vitals remained stable during the observation period. Repeat chest X-ray re-demonstrated the previously documented diffuse subcutaneous emphysema, pneumomediastinum, and pneumothorax. The patient's condition remained unchanged during his hospital course and was discharged home. He later followed up with his primary care provider five days following his hospital discharge for evaluation of any acute changes. At this time, the patient was still hemodynamically stable and saturating well on room air, without any complaints of shortness of breath. However, widespread crepitus was still palpated across the upper chest and back. The patient was then instructed to follow up with the pulmonology clinic and to seek the emergency department for any new or worsening symptoms.

## Discussion

Rare cases of extensive subcutaneous emphysema have been documented and most commonly occur following pneumothorax, penetrating trauma and rib fractures, barotrauma, or an iatrogenic insult [5,6,8]. More severe cases of this phenomenon are more often secondary to iatrogenicity, whereas mild cases are associated with pneumothoraces [1]. However, there have been no documented cases of purposeful body insufflation in the literature to our knowledge. Furthermore, in this patient, this inflation subsequently led to the cooccurrence of pneumoperitoneum, pneumomediastinum, pneumopericardium, and pneumothorax. While these conditions can manifest alongside one another in cases of subcutaneous emphysema, it is exceedingly rare for all to arise together at once, as in this case [9,10].

Since the subcutaneous fascia lacks significant barriers, the air is likely able to travel up to the chest, neck, and head without much difficulty [11]. However, once air infiltrates the peritoneal space and fascia, it can likely travel through minute fenestrations in the diaphragm to the mediastinum via the existing pressure gradient [10,12]. Communications between the peritoneum and mediastinum also exist in the fascial planes alongside the esophageal and aortic diaphragmatic hiatuses [11]. Additionally, there have been examples of individuals with undiagnosed defects in the diaphragm that may serve as channels for the movement of air past this barrier [12]. Although the exact mechanism is unclear, pneumopericardium may also ensue as air can gain access to the pericardium, possibly traveling along venous sheaths [13,14]. Once air enters the mediastinum, it may continue into the pleural space subsequently producing pneumothoraces, such as in our patient. Defects in the pleuroperitoneal membrane may also yield a direct connection between the peritoneum and pleural spaces as well [11].

While most cases of subcutaneous emphysema do not require treatment, dangerous complications such as trachea and blood vessel compression can result in respiratory compromise and skin necrosis, respectively [1,8]. Serious cases have even led to death in some circumstances, thus close observation of these patients is critical to prevent such outcomes [8]. And while several treatments have been proposed to alleviate diffuse subcutaneous emphysema, no definitive guidelines for the treatment of this unusual presentation exist outside of case reports [7]. Therefore, further investigation is required to both better understand the pathophysiology of these incidents and to possibly elucidate more decisive treatments for the management of these patients.

## Conclusions

Massive air infiltration into the peritoneal space leading to widespread subcutaneous emphysema and the subsequent movement of gas to adjacent body cavities is a rare but potentially life-threatening event. Careful observation and management of these patients, as well as the expansion of possible treatments, is crucial to prevent the development of serious complications. Ultimately, this case report highlights the consequences of large-volume self-inflicted peritoneal air insufflation and emphasizes the increased need for additional research into the management of these patients to reduce both morbidity and mortality.

## References

[1] Aghajanzadeh M, Dehnadi A, Ebrahimi H, Fallah Karkan M, Khajeh Jahromi S, Amir Maafi A, Aghajanzadeh G (2015). Classification and management of subcutaneous emphysema: a 10-year experience. Indian J Surg.

[2] Tran Q, Mizumoto R, Mehanna D (2018). Management of extensive surgical emphysema with subcutaneous drain: a case report. Int J Surg Case Rep.

[3] Srinivas R, Singh N, Agarwal R, Aggarwal AN (2007). Management of extensive subcutaneous emphysema and pneumomediastinum by micro-drainage: time for a re-think?. Singapore Med J.

[4] Park EY, Kwon JY, Kim KJ (2012). Carbon dioxide embolism during laparoscopic surgery. Yonsei Med J.

[5] Elkholy KO, Akhtar H, Landa E, Malyshev Y, Sahni S (2019). A case of pneumomediastinum and pneumoperitoneum with concurrent massive subcutaneous emphysema due to repositioning of a tracheostomy tube. Cureus.

[6] Kolleri JJ, Al-Warqi A, Mohamed RI, Khaliq A, Mirza S (2021). Extensive surgical emphysema following stab injury to the neck. Cureus.

[7] Maunder RJ, Pierson DJ, Hudson LD (1984). Subcutaneous and mediastinal emphysema: pathophysiology, diagnosis, and management. Arch Intern Med.

[8] Melhorn J, Davies HE (2021). The management of subcutaneous emphysema in pneumothorax: a literature review. Curr Pulmonol Rep.

[9] Fosi S, Giuricin V, Girardi V, Di Caprera E, Costanzo E, Di Trapano R, Simonetti G (2014). Subcutaneous emphysema, pneumomediastinum, pneumoretroperitoneum, and pneumoscrotum: unusual complications of acute perforated diverticulitis. Case Rep Radiol.

[10] Marwan K, Farmer KC, Varley C, Chapple KS (2007). Pneumothorax, pneumomediastinum, pneumoperitoneum, pneumoretroperitoneum and subcutaneous emphysema following diagnostic colonoscopy. Ann R Coll Surg Engl.

[11] Abdalla S, Gill R, Yusuf GT, Scarpinata R (2018). Anatomical and radiological considerations when colonic perforation leads to subcutaneous emphysema, pneumothoraces, pneumomediastinum, and mediastinal shift. Surg J (N Y).

[12] Zeno BR, Sahn SA (2006). Colonoscopy-associated pneumothorax: a case of tension pneumothorax and review of the literature. Am J Med Sci.

[13] Lee YJ, Jin SW, Jang SH (2001). A case of spontaneous pneumomediastinum and pneumopericardium in a young adult. Korean J Intern Med.

[14] Mansfield PB, Graham CB, Beckwith JB, Hall DG, Sauvage LR (1973). Pneumopericardium and pneumomediastinum in infants and children. J Pediatr Surg.

